# Lower respiratory tract microbial composition was diversified in *Pseudomonas aeruginosa* ventilator-associated pneumonia patients

**DOI:** 10.1186/s12931-018-0847-3

**Published:** 2018-07-27

**Authors:** Xiaoling Qi, Hongping Qu, Dandan Yang, Lian Zhou, Ya-Wen He, Yuetian Yu, Jieming Qu, Jialin Liu

**Affiliations:** 1 Department of Critical Care Medicine, Ruijin Hospital, Shanghai Jiao Tong University School of Medicine, Shanghai, China; 2 State Key Laboratory of Microbial Metabolism, School of Life Sciences & Biotechnology, Shanghai Jiao Tong University, Shanghai, China; 3 Department of Critical Care Medicine, Ren Ji Hospital, Shanghai Jiao Tong University School of Medicine, Shanghai, China; 4 Department of Pulmonary & Critical Care Medicine, Ruijin Hospital, Shanghai Jiao Tong University School of Medicine, Shanghai, China

**Keywords:** Pseudomonas aeruginosa, Mechanical ventilation, Microbiota, Lower respiratory tract, Prognosis, Intensive care unit

## Abstract

**Background:**

Probiotics could prevent *Pseudomonas aeruginosa* colonization in lower respiratory tract (LRT) and reduced *P. aeruginosa* ventilator-associated pneumonia (VAP) rate. Recent studies also suggested that probiotics could improve lung inflammation in mice infected with *P. aeruginosa.* It seems that microbiota regulation may be a potential therapy for *P. aeruginosa* VAP patients. However, we know less about the LRT microbial composition and its correlation with prognosis in *P. aeruginosa* VAP patients. This study aimed to characterize LRT microbiota in *P. aeruginosa* VAP patients and explore the relationship between microbiota and patient prognosis.

**Methods:**

Deep endotracheal secretions were sampled from subjects via intubation. Communities were identified by 16S ribosomal RNA gene sequencing. The relationship between microbiota and the prognosis of *P. aeruginosa* VAP patients were evaluated. Clinical pulmonary infection score and the survival of intensive care unit were both the indicators of patient prognosis.

**Results:**

In this study, the LRT microbial composition of *P. aeruginosa* VAP patients was significantly different from non-infected intubation patients, and showed significant individual differences, forming two clusters. According to the predominant phylum of each cluster, these two clusters were named Pro cluster and Fir-Bac cluster respectively. Patients from Pro cluster were dominated by *Proteobacteria* (adj.*P* < 0.001), while those from Fir-Bac cluster were dominated by *Firmicutes,* and *Bacteroidetes* (both adj.*P* < 0.001). These two varied clusters (Pro and Fir-Bac cluster) were associated with the patients’ primary disease (χ^2^-test, *P* < 0.0001). The primary disease of the Pro cluster mainly included gastrointestinal disease (63%), and the Fir-Bac cluster was predominantly respiratory disease (89%). During the two-week dynamic observation period, despite the use of antibiotics, the dominant genera and Shannon diversity of the LRT microbiota did not change significantly in patients with *P. aeruginosa* VAP. In prognostic analysis, we found a significant negative correlation between *Lactobacillus* and clinical pulmonary infection score on the day of diagnosis (*P* = 0.014); but we found no significant difference of microbial composition between survivors and non-survivors.

**Conclusions:**

LRT microbial composition was diversified among *P. aeruginosa* VAP patients, forming two clusters which were associated with the primary diseases of the patients.

**Electronic supplementary material:**

The online version of this article (10.1186/s12931-018-0847-3) contains supplementary material, which is available to authorized users.

## Background

Ventilator-associated pneumonia (VAP) is a frequent complication in patients requiring mechanical ventilation and the associated mortality ranges from 20 to 50% [1, 2]. *Pseudomonas aeruginosa* is one of the most common pathogens causing VAP and is independently associated with increased mortality; in China, it has been staying in the top three pathogens [3–7]. Antibiotic treatment is the primary method for managing *P. aeruginosa* VAP; however, it constitutes a risk factor for the development of multi-drug resistant *P. aeruginosa* [2]. Increasing drug resistance, especially in intensive care units (ICUs), could result in *P. aeruginosa* VAP becoming uncontrollable [8, 9].

Recent findings suggested that the lower respiratory tract (LRT) is inhabited by niche-specific microbiota and VAP occurs mainly when the micro-ecology balance is damaged [10–12]. Probiotics play a preventive role on VAP occurrence, especially the VAP induced by *P. aeruginosa* [13–15]. Probiotics pre-treated patients obtained a decreased risk of LRT colonization with *P. aeruginosa* [13, 15]. In addition, Khailova et al. found that *Lactobacillus rhamnosus* can decrease lung *P. aeruginosa* load and increase the survival rate of *P. aeruginosa* pneumoniae mice [16]. It indicated that microbiota regulation could have significant effect on *P. aeruginosa* infection. However, we know less about the characteristics of LRT microbial composition in *P. aeruginosa* VAP patients.

In this study, our aim was to examine LRT microbiota characteristics in *P. aeruginosa* VAP first and then analyze the relationship between LRT microbial characteristics and patient prognosis.

## Methods

### Subjects

This study was a prospective study conducted at intensive care unit (ICU) of Ruijin Hospital, China. Inclusion criteria of *P. aeruginosa* VAP patients included [1]: (1) mechanical ventilation > 48 h; (2) satisfied two of the following: body temperature > 38 °C or < 36 °C, leukopenia or leukocytosis, or purulent secretions; (3) new or progressive chest infiltrates, for patients with underlying pulmonary or cardiac disease, two serial chest radiographs were required for assessment; (4) endotracheal aspiration cultured *P. aeruginosa* at least + 2 growth using semi-quantitative measurements. Exclusion criteria included: (1) age < 18 years; (2) pregnant woman; (3) sputum cultured *P. aeruginosa* prior to intubation. Clinical data collection was performed at the hospital upon admission and terminated following study withdrawal, discharge, or death. Sequential Organ Failure Assessment (SOFA) score was evaluated at ICU admission and the initial sample collection day. The severity of pulmonary infection in *P. aeruginosa* VAP patients was assessed using the Clinical Pulmonary Infection Score (CPIS). The criteria of CPIS were performed as previously described [17]. CPIS could be used to assess the clinical outcome of pulmonary infection in patients with VAP [18]. We took the CPIS of the patient at the time of diagnosis as the baseline parameter and reassessed it within 7 days to 14 days after antibiotic treatment; the CPIS score was reduced to within 6 points to be recognized as clinical improvement of pulmonary infection [17]. Control subjects were selected from selective operation patients without acute or chronic respiratory disease, any infection, and antibiotic use for three months prior. Written informed consents were obtained from all study subjects or their lineal consanguinities prior to enrollment. The protocol of this study was approved by the Ruijin Hospital Ethics Committee Shanghai Jiao Tong University School of Medicine.

### Sample collection

Initial samples were collected within 24 h post *P. aeruginosa* VAP diagnosis. Sequential sample collection was performed at day 7 and day 14 post initial sample collection. Endotracheal aspiration samples were collected using an endotracheal tube. Collected samples were stored in sterile 15 mL centrifuge tubes at − 80 °C.

### Freeze-drying

We set up negative control using sterile deionized water. Prior to freeze-drying, the sample tubes were transferred into a portable liquid nitrogen container and the sample tubes caps were substituted with parafilm; five small holes were made using sterile yellow tips in a bio-safety cabinet while the sample tubes remained in liquid nitrogen. The sample shelf of the freeze-dryer (FreeZone 6 Liter Console Freeze Dry Systems, Labconco, USA) was pre-cooled for at least 2 h in advance by setting the trap-temperature to − 86 °C. The pre-treated frozen samples were then transferred into the sample shelf in the freeze-dryer (chamber space = 2.82 × 107 mm3, trap-temperature = − 86 °C, and vacuum pressure = 0.165 Torr) for drying. Samples were incubated for 60 h. The samples were hermetically sealed immediately following vacuum release and then stored at − 80 °C.

### Bacterial DNA amplification and sequencing

LRT bacterial DNA extraction from freeze-dried powder was performed as previously described [19], including negative controls which set up in the freeze-drying procedure. The V3-V4 region of 16S ribosomal RNA (16S rRNA) gene from each DNA sample was amplified with primers F1 and R2 (5′- CCTACGGGNGGCWGCAG-3′ and 5′-GACTACHVGGGTATCTAATCC-3′) corresponding to positions 341 to 805 of the *Escherichia coli* 16S rRNA gene using an EasyCycler 96 PCR system (Analytik Jena Corp, AG) with the following program: 3 min at 95 °C (denaturation); 21 cycles of 30 s at 94 °C (denaturation), 30 s at 58 °C (annealing), and 30 s at 72 °C (elongation); 5 min at 72 °C (final extension). The products from different samples were indexed and mixed at equal ratios for sequencing using the Miseq platform (Illumina Inc., USA) according to the manufacturer’s instructions.

### Data processing

In this study, negative control samples were not detected any bands when the DNA amplification process was completed. We also sequenced those negative control samples, and a total of 90 sequences were obtained from negative control samples, which can be classified as 31 genera. Within the 31 genera, only two (*Escherichia* and *Streptococcus*) belong to the contaminant genera detected in negative controls by previous study [20]. The *Escherichia* was not found in any particular sample in our study; *Streptococcus* was the commensal of LRT, and the average relative abundance was 9.7% in the samples from controls and 0.5% in the samples from *P. aeruginosa* VAP patients.

Raw FASTQ files were demultiplexed and quality-filtered using USEARCH 8.0 with the following criteria: (1) exact index matching, (2) only sequences with > 50 bp overlaps were assembled according to their overlap sequence, (3) merged sequences > 400 bp, and (4) a maximum mismatch in overlap area <0.1. Reads that could not be assembled were discarded. Operational taxonomic units (OTUs) were clustered with 97% similarity cutoff using UPARSE (version 7.1) after chimeric sequences removed. The phylogenetic affiliation of each 16S rRNA gene sequence was analyzed using RDP Classifier against the SILVA (SSU123) 16S rRNA database using a confidence threshold of 70%. The raw sequencing data have been uploaded in the NCBI GenBank Sequence Read Archive database (accession number SRP112361).

### Statistical analysis

To access the sequencing depth of the LRT samples, a rarefaction curve was generated as previous study [21] and it indicated that the sequencing depth of samples was reasonable (Additional file 1: Figure S1). Shannon’s diversity was calculated by Mothur. The significant difference in Shannon index between groups was calculated using the Wilcoxon nonparametric test. The unweighted UniFrac distance and weighted UniFrac distance were calculated using Quantitative Insights into Microbial Ecology (QIIME) to assess compositional dissimilarity between samples and finally showed as principal co-ordinates analysis (PCoA) plots conducted in R version 3.2.1 [22]. To find factors that related to the LRT microbial composition in *P. aeruginosa* VAP patients, patient samples from the initial collection day were subjected to a similarity-based, unsupervised hierarchical clustering [23, 24]. Clustering analysis was performed by unweighted pair-group method with arithmetic means (UPGMA) with Bray Curtis distance [23]. Spearman’s correlation was used to test the relationship between the relative abundance of each genus found in *P. aeruginosa* VAP patients and CPIS score, SOFA score. The statistic comparison of relative abundance of all taxa between the groups was analyzed using the Wilcoxon rank sum test and the *p*-values were calculated for the false discovery rate (FDR) (q value) [25]. In the dynamic analysis, the analysis of significant differences in Shannon diversity at different time points using paired nonparametric tests.

Clinical data were analyzed using SPSS version 23 (Armonk, New York, USA). Differences between groups were tested using a two-tailed t-test, Mann-Whitney U test, or chi-squared test as appropriate. Continuous variables were presented as the mean ± standard deviation for normally distributed data and median [interquartile range, IQR] for non-normally distributed data. Categorical variables were presented as number of subjects and percentages. Figures were created using GraphPad Prism version 6.0.

## Results

### Patient characteristics and sequencing

This study included 36 *P. aeruginosa* VAP patients and 18 control subjects. Sequential samples were obtained from 26 *P. aeruginosa* VAP patients for dynamics evaluation. Baseline characteristics are listed in Additional file 1: Table S1. Mean age, which was a known risk factors for VAP, was higher for *P. aeruginosa* VAP patients than for controls (*P* = 0.009). All *P. aeruginosa* VAP patients used antibiotics before mechanical ventilation. There was no significant difference in gender, body mass index (BMI), number of smokers, number of common complications including hypertension and diabetes in *P. aeruginosa* VAP group and control group (Additional file 1: Table S1).

One sample collected from *P. aeruginosa* VAP patient in this study failed to sequence, and we have excluded this sample. Finally, there were 94 samples for the further analysis. In total, 2,211,722 sequences were obtained from 94 samples (median 25,391; IQR 13,216–32,270 sequences per sample) by 16S rRNA gene sequencing. Within the 94 samples, 914 OTUs were detected and classified into 19 phyla including 269 genera.

### LRT microbiota profiles

**Fig. 1:**
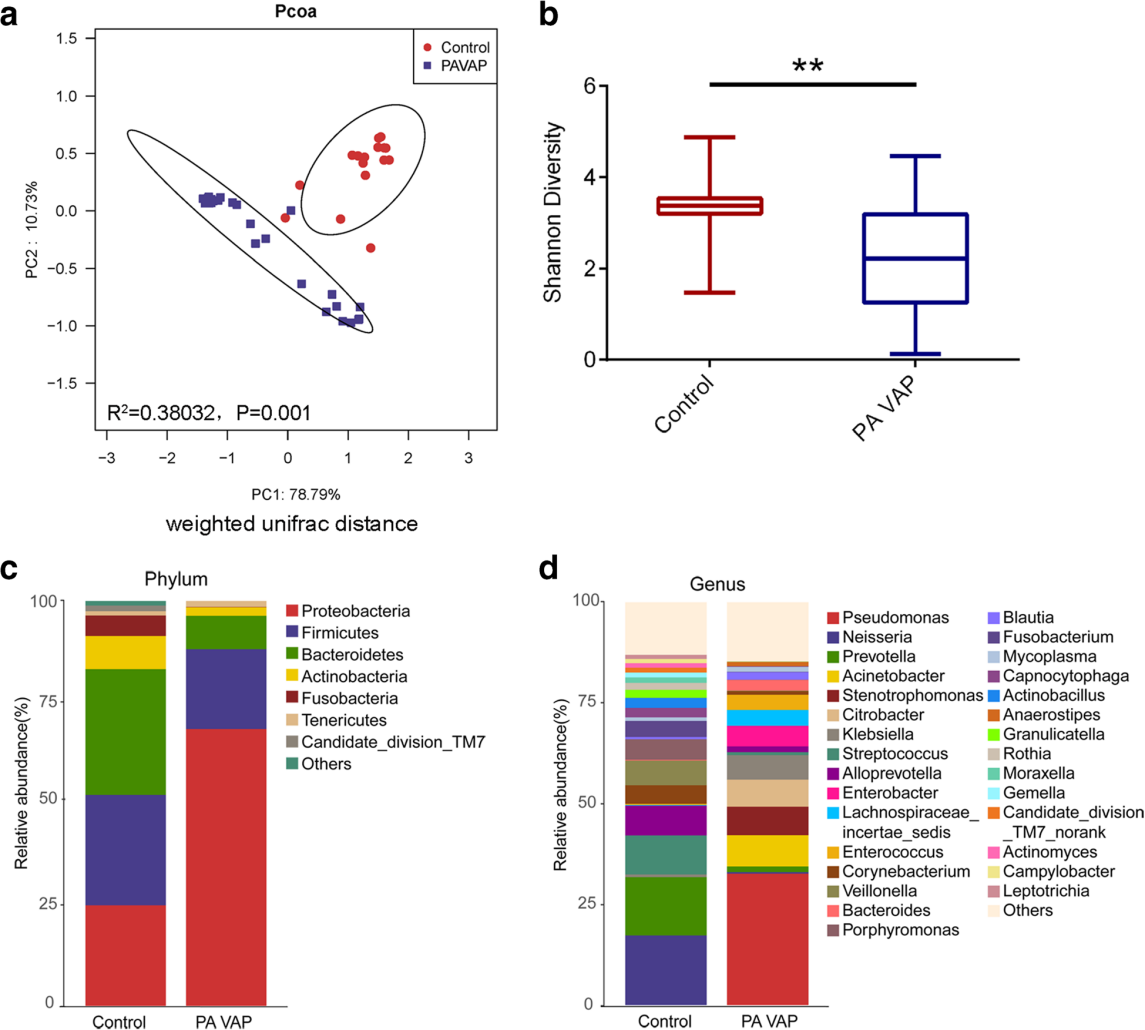
The LRT microbiota of *P. aeruginosa* VAP patients was significantly different from that of the controls. Samples from day1 were collected for analysis. **a** The weighted unifrac distance between the LRT microbiota of each sample shown as a Pcoa two-dimensional map. Each dot represents one sample. **b** Shannon diversity index of the *P. aeruginosa* VAP group was significantly lower than those in the control group. **c** and **d** Histogram of lower respiratory tract microbial composition at phylum level (**c**) and at genus level (**d**) in *P. aeruginosa* VAP patients and control subjects. Data were presented as mean ± Standard deviation. The difference between groups was analyzed using the Mann-Whitney test, **P < 0.01. Control, control group; PAVAP, Pseudomonas aeruginosa VAP group

Based on the phylogenetic relationships among the members of the microbial community, the similarity of the LRT microbial composition was measured by the unifac distance. It was found that the composition of the LRT microbiota in patients with *P. aeruginosa* VAP was significantly different from that in the control group (unweighted unifrac distance, R^2^ = 0.18431, *P* = 0.001; weighted unifrac distance, R^2^ = 0.38032, P = 0.001) (Fig. 1a; Additional file 1: Figure S2). Diversity, presented as Shannon index, was lower in patients with *P. aeruginosa* VAP compared to the controls (*P* = 0.0025) (Fig. 1b).

To obtain a global view of LRT microbiota in study subjects, we compared the taxa at phylum and genus level between control and *P. aeruginosa* VAP group. At phylum level, the dominant taxa of patients in the control group and the *P. aeruginosa* group were both *Proteobacteria*, *Firmicutes*, and *Bacteroidetes*, but the average abundance of *Proteobacteria* in the *P. aeruginosa* group was significantly increased (adj.*P* = 3.02 × 10^− 5^) (Fig. 1c). At the genus level, patients in the control group were dominated by *Neisseria*, *Prevotella*, *Streptococcus* and *Alloprevotella*, while *Pseudomonas*, *Acinetobacter*, *Enterobacteriaceae* (*Citrobacter*, *Klebsiella*, *Enterobacter*) and *Lachnospiraceae_incertae_sedis* were dominant taxa in the *P. aeruginosa* group (Fig. 1d).

### Primary disease related to LRT microbial clustering of *P. aeruginosa* VAP patients

**Fig. 2:**
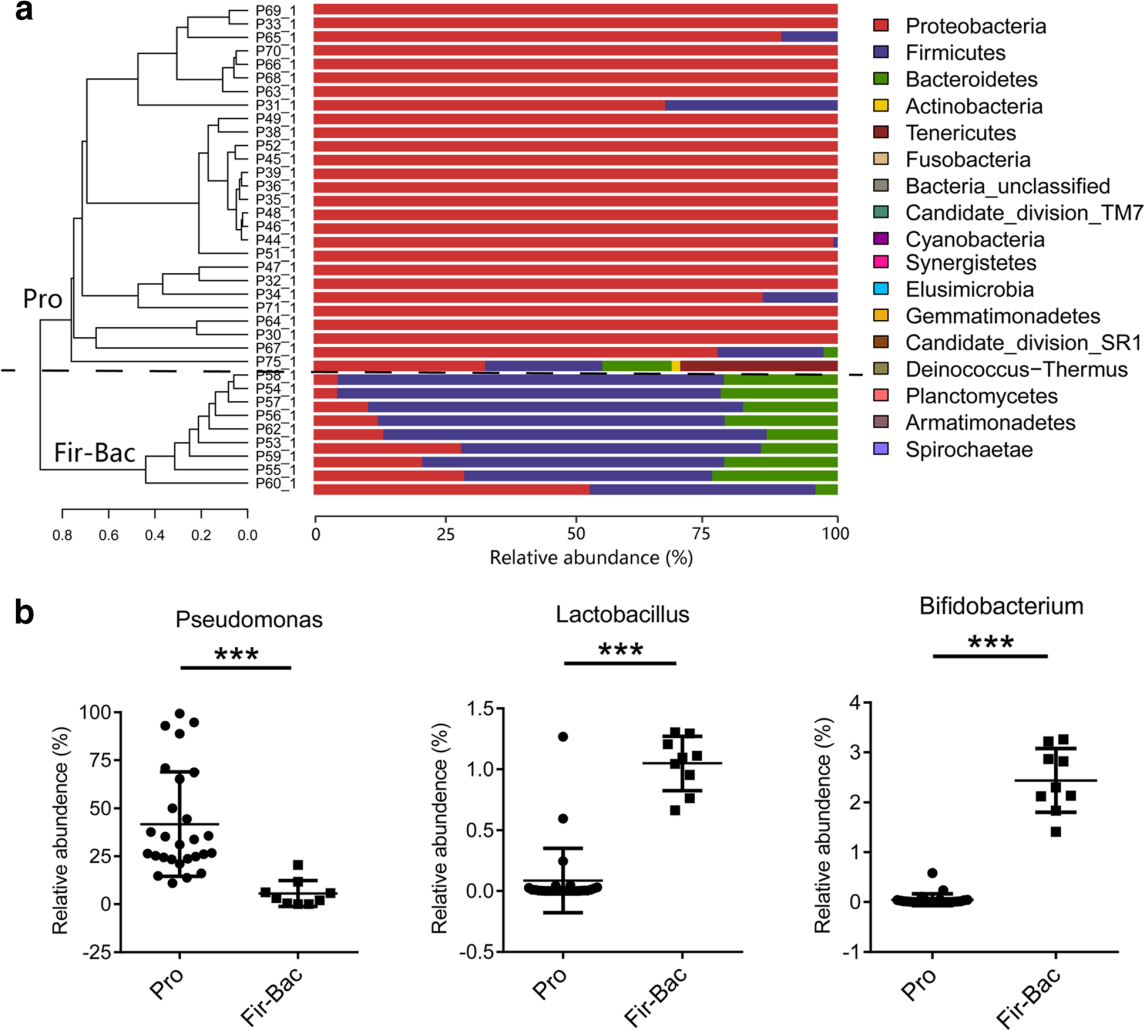
The LRT microbial composition and hierarchical clustering of *P. aeruginosa* VAP patients. Initial samples were analyzed. **a** The Bray curtis distance matrix was used to construct a dendrogram including 36 *P. aeruginosa* VAP patients. Clusters were separated by black dotted line originating from the top of the dendrogram. The microbial composition at phylum level was visualized as bar charts. Patient number is indicated at the left of each bar. Each bar represents 100% of the OTUs detected per patient; OTUs are color-coded according to phylum. The predominant phylum in Pro cluster was *Proteobacteria,* and the predominant phylum in Fir-Bac cluster was *Firmicutes* and *Bacteroidetes.* **b** Comparison of different taxa between Pro cluster and Fir-Bac cluster. Data were presented as mean ± Standard deviation. The difference between groups was analyzed using the Mann-Whitney test, ***P < 0.001. LRT lower respiratory tract; VAP, ventilator-associated pneumonia; OTUs, operational taxonomic units

Meanwhile, we found microbial composition within *P. aeruginosa* VAP group varied; thus, we performed clustering analysis. Samples from *P. aeruginosa* VAP patients were hierarchically clustered based on similarities in microbial composition (measured by Bray Curtis distance) and visualized as phylum and genus dendrograms with two clusters (Fig. 2a; Additional file 1: Figure S3). Each cluster was given a name according to the most abundant taxon at phylum level and these two clusters were finally termed Pro cluster and Fir-Bac cluster. Pro cluster exhibited a high abundance of *Proteobacteria* at the phylum level (Fig. 2a) and *Pseudomonas*, *Citrobacter*, *Enterobacter*, *Klebsiella*, *Enterococcus* and *Acinetobacter* at the genus level (Additional file 1: Figure S3). The taxon composition of Fir-Bac cluster consisted mainly of *Firmicutes* and *Bacteroidetes* at the phylum level (Fig. 2a) and *Lachnospiraceae_incertae_sedis, Bacteroides*, *Blautia*, and *Alloprevotella* at the genus level (Additional file 1: Figure S3). In addition, we found the abundance of *Bifidobacterium* and *Lactobacillus* in Fir-Bac cluster were significantly higher than those of the Pro group (both adj.*P* < 0.0001), whereas the abundance of *Pseudomonas* was significantly lower than that of Pro group (adj.*P* < 0.001) (Fig. 2b). Conventional culture of endotracheal aspiration samples was also obtained from *P. aeruginosa* VAP patients; the pathogens cultured by conventional methods were accordant with the 16S rRNA sequence data in most of patients (Additional file 1: Table S2, Additional file 1: Figure S3).

Interestingly, the primary diseases between Pro cluster and Fir-Bac cluster were significantly different. Patients from Pro cluster were mainly suffer from extra-lung disease while patient from Fir-Bac cluster were mainly suffer from lung disease (χ^2^-test P < 0.0001) (Table 1). Other baseline parameters, including age, body mass index, smoking, chronic coexistence of disease, sepsis, disease severity and the occurrence time of *P. aeruginosa* VAP, showed no significant difference between Pro cluster and Fir-Bac cluster (Table 1). Disease severity was assessed using Acute Physiology and Chronic Health Evaluation (APACHE) II scores and SOFA scores upon ICU admission.

**Table 1:** Baseline characteristics of *P. aeruginosa* VAP patients classified by LRT microbial similarity

Characteristics	Pro cluster (*n* = 27)	Fir-Bac cluster (*n* = 9)	*P* value
Demographics			
Age, y	67.74 ± 14.55	69.78 ± 12.44	0.709
Gender, female, *n* (%)	9 (33%)	6 (67%)	0.122
BMI, kg/m^2^	23.45 ± 4.31	21.00 ± 4.40	0.152
Smoker, *n* (%)	6(22%)	0 (0%)	0.303
Primary disease			
Respiratory disease^a^, *n* (%)	2(7%)	8 (89%)	< 0.0001
Gastrointestinal disease^b^, *n* (%)	17 (63%)	1 (11%)	
Neurological disease, *n* (%)	4 (15%)	0 (0%)	
Trauma, *n* (%)	2 (7%)	0 (0%)	
Bone and joint damage, *n* (%)	1 (4%)	0 (0%)	
Autoimmune disease, *n* (%)	1 (4%)	0 (0%)	
Chronic coexisting disease			
Chronic bronchitis	3(11%)	1(11%)	1.000
Chronic obstructive pulmonary disease	2 (7%)	0(0%)	1.000
Diabetes, *n* (%)	3 (11%)	3 (33%)	0.151
Hypertension, *n* (%)	12 (44%)	4 (44%)	1.000
Disease severity			
APACHE II^c^	18 [16,24]	16 [9.5–24]	0.233
SOFA^c^	8[2.5,10]	5[3,7]	0.263
Diagnosis of sepsis prior to sampling, n (%)	15 (55%)	6 (67%)	0.705
Time of *P. aeruginosa* VAP onset, day^d^	15 [7–24]	14 [2–60.5]	0.971

Abbreviations: *VAP* ventilator-associated pneumonia, *BMI* body mass index, *APACHE II* Acute Physiology and Chronic Health Evaluation II, *SOFA* Sequential Organ Failure Assessment

a. 7 patients with severe pneumonia (not induced by *P. aeruginosa*), and 1 patient with pharyngeal abscess in *Fir-Bac* cluster and one with acute exacerbation of chronic obstructive pulmonary disease, and one with hypopharyngeal carcinoma in *Pro* cluster

b. One patient with acute obstructive suppurative cholangitis in *Fir-Bac* cluster and 4 with gastrointestinal tumors, 6 with acute pancreatitis, 1 with bile duct stones, 1 with bile duct obstruction, 3 with intestinal obstruction and 2 with gastrointestinal perforation in *Pro* cluster

c. APACHE II and SOFA scores were calculated within the first 24 h of ICU admission

d. Time of *P. aeruginosa* VAP onset was defined as the interval from endotracheal intubation to diagnosis of *P. aeruginosa* VAP

### No effect of antibiotics on LRT microbiota in *P. aeruginosa* VAP patients

Previous studies have suggested that antibiotic was an important factor affecting the microbial composition, so we would explore whether antibiotics are associated with microbial clustering. All patients in this study had been treated with antibiotics prior to sampling in *P. aeruginosa* VAP patients. Additional file 1: Table S2 showed the use of antibiotics for 36 *P. aeruginosa* VAP patients one week before sampling. We found that there were no statistically significant differences in aminoglycosides, carbapenems, cephalosporins, fluoroquinolones, and vancomycin in the Pro cluster and the Fir-Bac cluster, and the number of antibiotics used (all *P* > 0.05) (Additional file 1: Table S4).

### The LRT microbiota of *P. aeruginosa* VAP patients was associated with the severity of VAP

To gain insight into correlation between LRT microbiota and severity of pulmonary infection (measured by CPIS) in patients with *P. aeruginosa* VAP, we performed Spearman correlation analysis between all genera and CPIS that collected within 24 h post *P. aeruginosa* VAP diagnosis. It was found that 21 genera showed significantly positive correlation with CPIS while 47 genera showed significantly negative correlation with CPIS (Table 2 and Table 3). Among the genera that showed a significant positive correlation with CPIS in *P. aeruginosa* VAP patients, *Burkholderia*, *Alcaligenes*, *Pseudomonas*, *Massilia*, *Flavobacterium* and *Enterobacter* can cause lung infections, *Sphingomonas* is a pathogen that can cause wound infections in animals, and *Methylobacterium* is often isolated in the hospital environment (all *P* < 0.05) (Table 2). *Lactobacillus* and *Bifidobacterium* were both negatively correlated with CPIS in patients with *P. aeruginosa* VAP, but there was no statistically significant difference between *Bifidobacterium* and CPIS (*Lactobacillus*: *R* = − 0.4024, *P* = 0.014; *Bifidobacterium*: *R* = − 0.3096, *P* = 0.066) (Table 3).

**Table 2:** Positive correlation between genus of *P. aeruginosa* VAP and CPIS

Genus	R	P
Burkholderia	0.517183	0.001238
Acidaminobacter	0.483879	0.00279
Alcaligenes	0.441525	0.007023
Methylocella	0.432106	0.008495
Sphingomonas	0.42808	0.009199
Acidisoma	0.426819	0.00943
Curvibacter	0.422908	0.010177
Pseudomonas	0.41731	0.011335
Dysgonomonas	0.400763	0.015426
Gemmata	0.395769	0.016881
Macellibacteroides	0.392997	0.017737
Diaphorobacter	0.39107	0.018354
Brevundimonas	0.383566	0.020927
Massilia	0.378151	0.022965
Flavobacterium	0.37017	0.026267
Janthinobacterium	0.368243	0.02712
Methylobacterium	0.367719	0.027356
Petrimonas	0.366787	0.02778
Enterobacter	0.360325	0.030869
Azotobacter	0.349351	0.036758
Reyranella	0.332149	0.047799

**Table 3:** Negetive correlation between genus of *P. aeruginosa* VAP and CPIS

Genus	R	P	Genus	R	P
Megamonas	−0.584	0.00018	Sutterella	− 0.404	0.01465
Anaerovorax	− 0.554	0.00046	Lactobacillus	− 0.402	0.01497
Intestinimonas	− 0.511	0.00146	Phascolarctobacterium	−0.394	0.01744
Sporosarcina	−0.502	0.00180	Alloprevotella	−0.394	0.01750
Thalassospira	−0.495	0.00214	Ruminococcus	−0.392	0.01813
Mitsuokella	−0.495	0.00214	Holdemania	−0.391	0.01822
Marvinbryantia	−0.495	0.00217	Butyrivibrio	−0.391	0.01825
Jeotgalicoccus	−0.494	0.00220	Paraprevotella	−0.384	0.02070
Coprobacillus	−0.491	0.00234	Blautia	−0.377	0.02330
Howardella	−0.490	0.00239	Erysipelotrichaceae_incertae_sedis	−0.374	0.02458
Catenibacterium	−0.482	0.00292	Peptococcus	−0.372	0.02530
Coprobacter	−0.480	0.00305	Candidatus_Saccharimonas	−0.370	0.02591
Facklamia	−0.466	0.00414	Lachnospiraceae_incertae_sedis	−0.366	0.02825
Aeriscardovia	−0.466	0.00417	Ruminococcaceae_incertae_sedis	−0.364	0.02904
Dorea	−0.444	0.00668	WCHB1-69_norank	−0.357	0.03240
Corynebacterium	−0.441	0.00706	Clostridium_sensu_stricto_1	−0.355	0.03355
Bacteroides	−0.438	0.00752	Acetatifactor	−0.350	0.03666
Oscillibacter	−0.432	0.00848	Aerococcus	−0.348	0.03727
Atopostipes	−0.431	0.00867	Coprococcus	−0.348	0.03759
Roseburia	−0.425	0.0098	Anaerotruncus	−0.342	0.04151
Subdoligranulum	−0.421	0.01053	Family_XIII_incertae_sedis	−0.341	0.04155
Alistipes	−0.421	0.01059	Megasphaera	−0.339	0.04295
Acidaminococcus	−0.409	0.01318	RF9_norank	−0.337	0.04455
Anaerostipes	−0.409	0.01323	Bifidobacterium	−0.310	0.06610

### LRT microbiota may not be related to the survival of the ICU in *P. aeruginosa* VAP patients

**Fig. 3:**
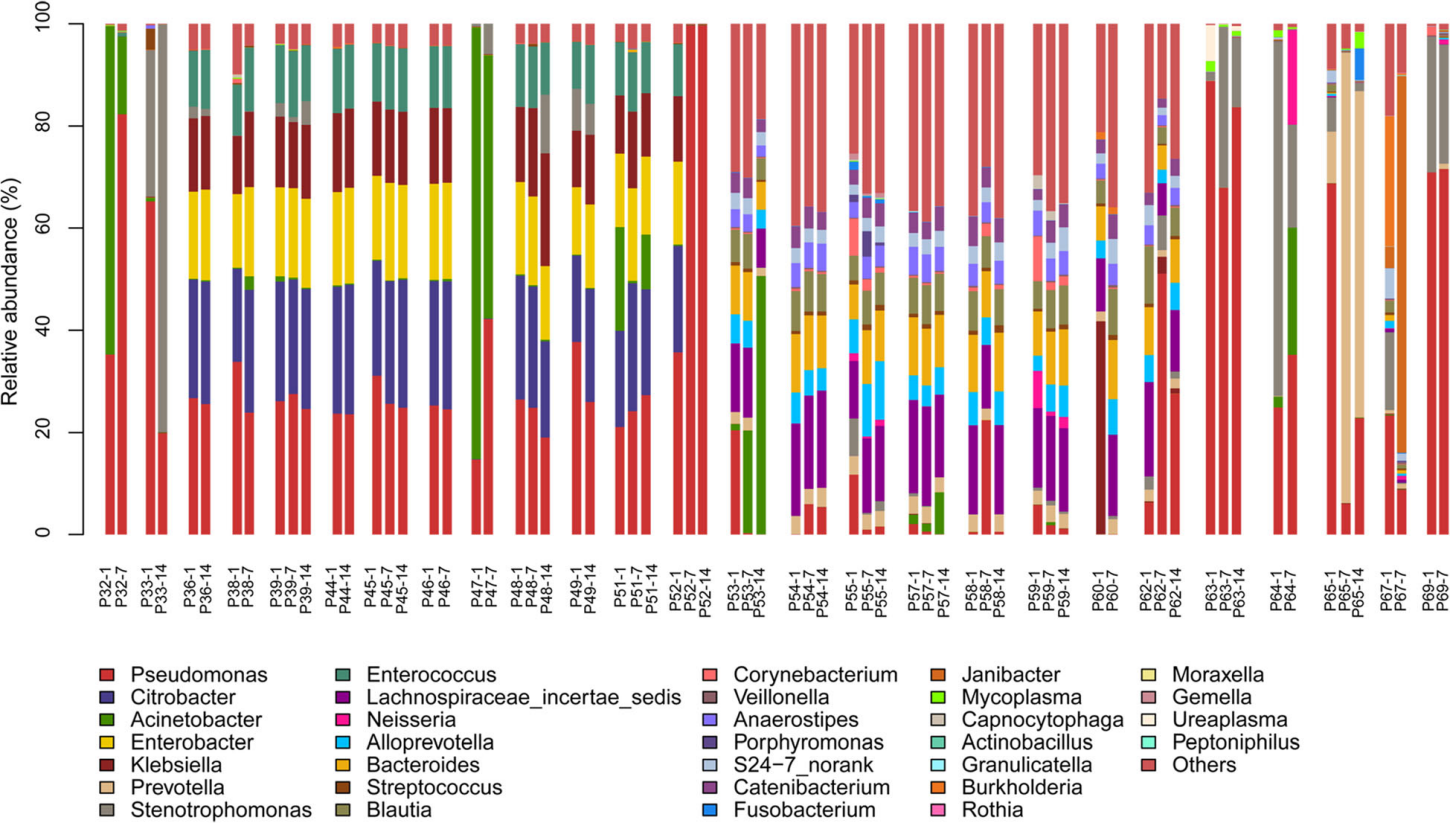
Dynamic variation of LRT microbiota in *P. aeruginosa* VAP patients two weeks post initial sample collection. Samples of 26 *P. aeruginosa* VAP patients collected on day 1, day 7, and day 14 were analyzed. Day 1 designates the initial sample collected. Microbial composition is presented as a histogram with patient number and collection time indicated at the bottom of bars. Each bar represents 100% of the OTUs detected per patient and the OTUs are color-coded according to genus. Only genera with a frequency > 5% were included. VAP, ventilator-associated pneumonia; OTUs, operational taxonomic units

40 sequential samples collected from patients with *P. aeruginosa* VAP on days 1, 7, and 14 (day 1 was the initial sample collection day). Those patients were all received antibiotics during the two-week observation (Additional file 1: Table S5). The Shannon diversity was relatively stable from day 1 to day 7 and day 1 to day 14 in most *P. aeruginosa* VAP patients (Additional file 1: Figure S4). The predominant species was stable over time regardless of antibiotic treatment and *Pseudomonas* proportion fluctuated in nearly all patients (Fig. 3). During the two-week observation, there was no genera changed (paired Wilcoxon test, all adj.*P* > 0.05).

During the two-week observation, six patients with *P. aeruginosa* VAP obtained clinical improvement. No significant taxa was found when we compared the microbial composition between acute phase and clinical improvement phase in those patients (Wilcoxon test of all taxa was adj.P > 0.05). However, of the six patients with *P. aeruginosa* VAP, abundance of *Pseudomonas* was decreased in five patients while was increased in one patients (from 21 to 24%) (Additional file 1: Figure S5). We also observed that *Lactobacillus* was increased in four *P. aeruginosa* VAP patients and *Bifidobacterium* was increased in five *P. aeruginosa* VAP patients (Additional file 1: Figure S5).

**Fig. 4:**
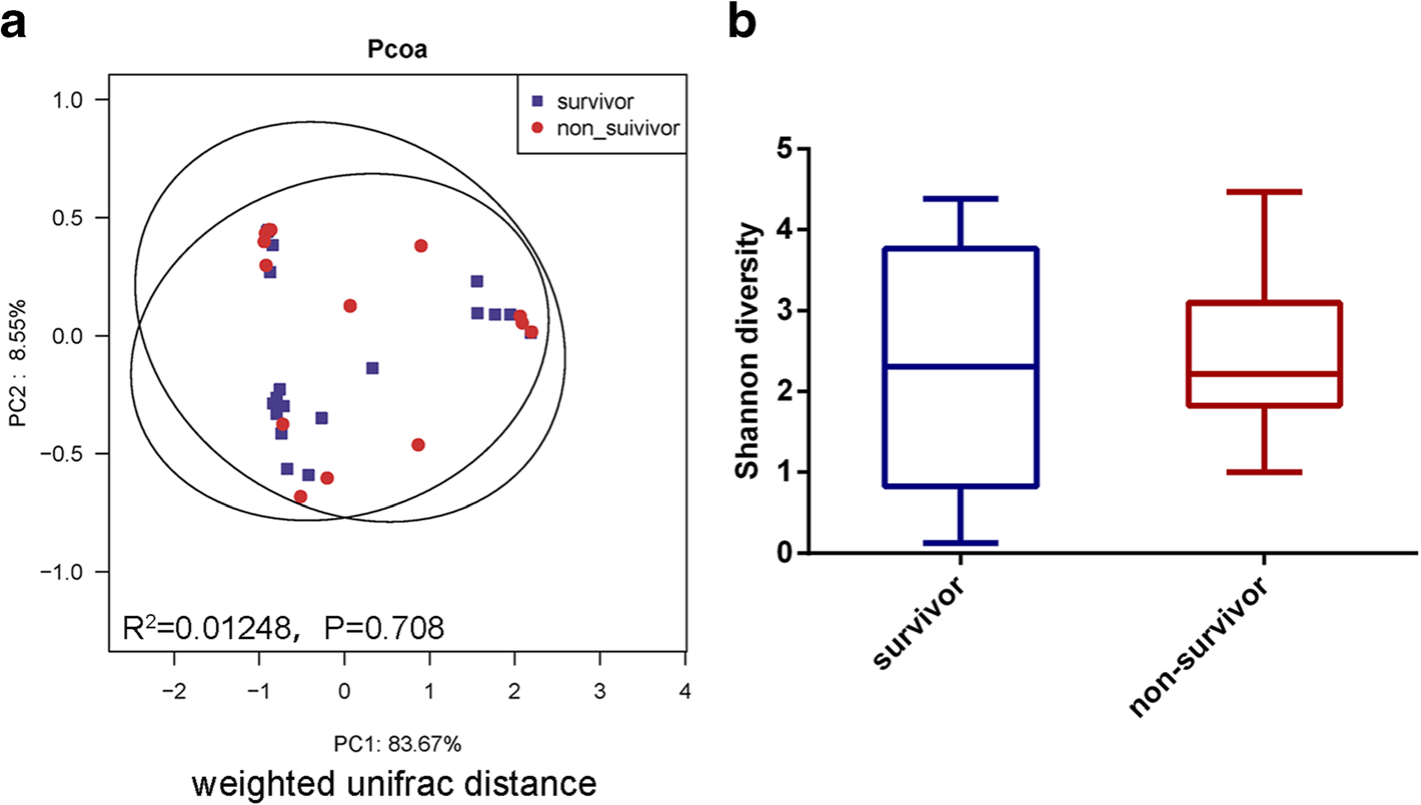
The LRT microbiota of survivors of *P. aeruginosa* VAP patients had no significant difference compared to that of the non-survivors. Samples from day1 were collected for analysis. **a** The weighted unifrac distance between the survival group and non-survival group shown as a Pcoa two-dimensional map. Each dot represents one sample. **b** Shannon diversity index had no statistical difference between survival group and non-survival group (*P* > 0.05). Data were presented as mean ± Standard deviation. The difference between groups was analyzed using the Mann-Whitney test

In order to clarify the relationship between LRT microbiota and survival of *P. aeruginosa* VAP patients, we first performed a Spearman correlation analysis between the relative abundance of genera and SOFA score evaluated in the initial sample collection day. We found that only 4 genera (WCHB1-69_norank, *Sphingopyxis*, *Reyranella* and *Sphingobacterium*) had significant negative correlation with SOFA score (Additional file 1: Table S5). No genus that had significant positive correlation  with the SOFA score was found. Next, we divided the *P. aeruginosa* VAP patients into survival group and non-survival group at the time of discharge. Of the 16 non-survivors, 12 belonged to *Pro* group and 4 belonged to *Fir-Bac* group (*Pro* group vs. *Fir-Bac* group, χ^2^-test P > 0.05). The LRT microbial composition and Shannon diversity of the survivors and non-survivors showed no significant difference (unweighted Unifrac distance, R^2^ = 0.02573, *P* = 0.468; weighted Unifrac distance, R^2^ = 0.01248, *P* = 0.708; Shannon index, *P* = 0.689) (Fig. 4; Additional file 1: Figure S6).

## Discussion

In this study, we described the dynamic characteristics of the LRT microbiota in patients with *P. aeruginosa* VAP and their association with clinical characteristics and severity of pneumonia. Microbial composition varied among *P. aeruginosa* VAP patients, forming two clusters. The microbial clusters related to the primary diseases of *P. aeruginosa* VAP patients. We also analyzed the correlation between microbial composition and the severity of pneumonia in *P. aeruginosa* VAP patients. We found that abundance of *Pseudomonas* was positively correlated with the severity of pneumonia, while the abundance of *Lactobacillus* was negatively correlated with the severity of pneumonia. .

We found *P. aeruginosa* VAP patient samples were depleted for LRT commensal bacteria (*Streptococcus* and *Veillonella*) and the predominant taxa differed by cluster. Fir-Bac cluster was enriched for *Lachnospiraceae_incertae_sedis, Bacteroides*, *Blautia*, and *Alloprevotella*, and Pro cluster was enriched for *Proteobacteria* including *Enterobacteriaceae* and *Pseudomonas*. *Lachnospiraceae_incertae_sedis, Bacteroides*, and *Blautia* are the predominant (and harmless) taxa in normal healthy intestines [26]. *Enterobacteriaceae* and *Pseudomonas* are common opportunistic pathogens causing lung infections [2]. Microbial clusters of the LRT microbiota in patients with *P. aeruginosa* VAP appear to be related to the primary disease. The primary disease in the Pro group was mostly digestive diseases, whereas in the Fir-Bac cluster the patients were mainly respiratory diseases. Previous studies have shown that *Lachnospiraceae* and *Bacteroides* are the predominant taxa in fecal samples of patients with respiratory disease such as cystic fibrosis [27], while *Enterobacteriaceae* increases more frequently in the gut of patients with gastrointestinal diseases [28–30]. Intestinal microbiota can translocate into the lung and that increased intestine permeability is the most probable mechanism underlying microbiota translocation [31]. Few studies have focused on the relationship between LRT microbiota and different primary diseases in patients with hospital infection. There was one study suggested that the predominant taxa in patients with pulmonary sepsis were different from that of the abdominal sepsis [29]. Thus, our findings suggested that the variation in LRT microbiota may be related to the intestinal microbiota of *P. aeruginosa* VAP patients. However, further analysis of the intestinal microbiota of *P. aeruginosa* VAP patients is needed.

The CPIS is commonly used to assess the severity of lung infections in patients and the efficacy of antibiotic therapy [17, 18]. In this study, we found that *Burkholderia*, *Pseudomonas*, and *Enterobacter* were positively correlated with CPIS, while *Lactobacillus* was negatively correlated with CPIS. These results suggested that the LRT microbiota in patients with *P. aeruginosa* VAP was associated with the severity of pneumonia. We also found that *Pseudomonas* showed a decreasing trend in the patients that acquired clinical improvement, and *Lactobacillus* and *Bifidobacterium* showed an upward trend in those patients. *Burkholderia*, *Pseudomonas*, and *Enterobacter* were common pathogens of hospital-acquired pneumonia [2]. Although they are also present in normal respiratory tract, their abundance is extremely low, and their abundance increases are likely to exacerbate the severity of pneumonia [10, 32]. *Lactobacillus* and *Bifidobacterium* could inhibit pathogen proliferation, affect the behavior of other taxa, and regulate the host immune response [33]. Several studies have shown that *Lactobacillus* and *Bifidobacterium* have an inhibitory effect on *P. aeruginosa, Escherichia coli, Klebsiella pneumonia,* and *Enterococcus faecalis* in vitro [33–36]. Cotar et al. found that *Lactobacillus* decreases *P. aeruginosa* virulence by decreasing the expression of *lasI*, *lasR*, *rhlI*, and *rhlR* involved in quorum sensing and inhibition of biofilm formation [37]. It has also been demonstrated that *Bifidobacterium* has the ability to inhibit the adhesion of *P. aeruginosa* to epithelial cells [34]. Therefore, their decline was likely to exacerbate the severity of pneumonia in *P. aeruginosa* VAP patients. Microbiota regulation may become a potential therapy for *P. aeruginosa* VAP, however, the issues of delivery and probiotic selection require careful consideration. Although we found LRT microbiota was associated with the severity of pneumonia and early treatment effect, we did not observed difference between survival group and non-survival group. The sampling collection time was far from the death time, ranged from one month to 12 months, which may be one of the reasons that resulted in no significant difference in the composition of the LRT microbial composition between the survivors and non-survivors. On the other hand, we found the SOFA score which was associated with patients’ survival showed little correlation with LRT microbiota in *P. aeruginosa* VAP patients. It seemed that the LRT microbiota had greater effect on lung than the whole organ system.

Though antibiotic species and numbers were individual in each *P. aeruginosa* VAP patient, patients of each cluster showed similar microbial composition. Our findings were similar to previous lung-infection-disease studies which showed the lung dominant taxa were stable following the antibiotic treatment [38, 39]. Rogers et al. found that long-term antibiotic therapy did not changed the lung microbial composition in non-cystic fibrosis bronchiectasis patients with baseline airway dominated by *P. aeruginosa* but changed in those not dominated by *P. aeruginosa* [40]. The mechanisms were complicated: competitions between bacterial species and antibiotic-bacterial interactions may involve in it. *Pseudomonas, Enterobacteriaceae* and *Acinetobacter* were common antibiotic-tolerant pathogens; it was not surprising that they could exist consistently with antibiotic exposure. The consistent microbial dysbiosis had significant negative impact on patient health, so identifying community member interactions and how to break up the network should be pay attention in future studies.

This study had several limitations. First, the sample size was limited. We could not exclude other microbial types that may exist in *P. aeruginosa* VAP. Second, we characterized the LRT microbiota using the culture-independent technology, which could identify low-proportion or uncultured taxa; however, we were unable to determine the function of specific species accurately. Further analysis regarding probiotic species and associated mechanisms in *P. aeruginosa* VAP is needed. Third, in this study, the sample collection time was limited. If we could increase the collecting time points in future, it may be better to reflect the changes of the LRT microbiota of patients with *P. aeruginosa* VAP.

## Conclusions

In summary, we described the composition and diversity of the LRT microbiota in patients with *P. aeruginosa* VAP. Importantly, we have found that in those *P. aeruginosa* VAP patients, the microbial composition of the LRT is diversified; and that difference was associated with the type of primary disease. We also found that the microbial composition of LRT was related to the severity of *P. aeruginosa* VAP. Therefore, the LRT microbiota may be a potential target for the treatment of *P. aeruginosa* VAP.

## Additional file

Additional file 1: **Figure S1.** Rarefaction curve of samples collected from *P. aeruginosa* VAP patients and control subjects. Control, control group; PA VAP, Pseudomonas aeruginosa VAP group. **Figure S2.** The LRT microbiota was different between *P. aeruginosa* VAP patients and control subjects. The unweighted unifrac distance between the LRT microbiota of each sample was calculated and it was shown as a Pcoa two-dimensional map. Each dot represents one sample. Control, control group; PAVAP, Pseudomonas aeruginosa VAP group. **Figure S3.** The LRT microbiota and hierarchical clustering of *P. aeruginosa* VAP patients at genus level. Initial samples were analyzed. The Bray curtis distance matrix (in genus level) was used to construct a dendrogram including 36 *P. aeruginosa* VAP patients. Clusters were separated by black dotted line originating from the top of the dendrogram. The microbial composition at genus level was visualized as bar charts. Patient number is indicated at the left of each bar. Each bar represents 100% of the OTUs detected per patient; OTUs are color-coded according to genus. LRT lower respiratory tract; VAP, ventilator-associated pneumonia; OTUs, operational taxonomic units. **Figure S4.** The Shannon diversity between different time points was stable in LRT samples from *P. aeruginosa* VAP patients. Comparison of the Shannon diversity between day1 (initial samples) and day7 (A), day1 and day14 (B) were conducted by paired non-parametric test (both *P* > 0.05). **Figure S5.** The variety of the relative abundance of Pseudomonas, Lactobacillus and Bifidobacterium in acute phase and improvement phase of *P. aeruginosa* VAP patients. Pseudomonas (A) showed a decreased trend while Lactobacillus (B) and Bifidobacterium (C) showed an increased trend when the *P. aeruginosa* VAP patients obtained clinical improvement. **Figure S6.** The LRT microbiota of survivors of *P. aeruginosa* VAP patients had no significant difference compared to that of the non-survivors. Samples from day1 were collected for analysis. The unweighted unifrac distance between the survivors and non-survivors was calculated and it was shown as a Pcoa two-dimensional map. Each dot represents one sample. **Table S1.** Baseline characteristics of control subjects and *P. aeruginosa* VAP patients. **Table S2.** Conventional culture of endotracheal aspiration samples from *P. aeruginosa* VAP patients. **Table S3.** Antibiotic use before one week sampling of *P. aeruginosa* VAP patients. **Table S4.** Antibiotics statistics of *P. aeruginosa* VAP patients before sampling. **Table S5.** Antibiotic use during the dynamic observation period of *P. aeruginosa* VAP patients. **Table S6.** Negative correlation between genus of *P. aeruginosa* VAP and SOFA score. (ZIP 1314 kb)

## Abbreviations

APACHE: Acute Physiology and Chronic Health Evaluation
BMI: Body mass index
CPIS: Clinical Pulmonary Infection Score
FDR: False discovery rate
ICU: Intensive care unit
IQR: Interquartile range
LRT: lower respiratory tract
OTU: Operational taxonomic unit
PCoA: Principal co-ordinates analysis
SOFA: Sequential Organ Failure Assessment
UPGMA: Unweighted pair-group method witharithmetic means
VAP: Ventilator-associated pneumonia
