# Supplementary material for: Lower respiratory tract microbial composition was diversified in Pseudomonas aeruginosa ventilator-associated pneumonia patients
Source: Respir Res. 2018 Jul 27;19:139. doi: 10.1186/s12931-018-0847-3 (PMC6062970; doi:10.1186/s12931-018-0847-3)
Supplement: Supplementary file 1 — Figure S1. Rarefaction curve of samples collected from P. aeruginosa VAP patients and control subjects. Control, control group; PA VAP, Pseudomonas aeruginosa VAP group. Figure S2. The LRT microbiota was different between P. aeruginosa VAP patients and control subjects. The unweighted unifrac distance between the LRT microbiota of each sample was calculated and it was shown as a Pcoa two-dimensional map. Each dot represents one sample. Control, control group; PAVAP, Pseudomonas aeruginosa VAP group. Figure S3. The LRT microbiota and hierarchical clustering of P. aeruginosa VAP patients at genus level. Initial samples were analyzed. The Bray curtis distance matrix (in genus level) was used to construct a dendrogram including 36 P. aeruginosa VAP patients. Clusters were separated by black dotted line originating from the top of the dendrogram. The microbial composition at genus level was visualized as bar charts. Patient number is indicated at the left of each bar. Each bar represents 100% of the OTUs detected per patient; OTUs are color-coded according to genus. LRT lower respiratory tract; VAP, ventilator-associated pneumonia; OTUs, operational taxonomic units. Figure S4. The Shannon diversity between different time points was stable in LRT samples from P. aeruginosa VAP patients. Comparison of the Shannon diversity between day1 (initial samples) and day7 (A), day1 and day14 (B) were conducted by paired non-parametric test (both P > 0.05). Figure S5. The variety of the relative abundance of Pseudomonas, Lactobacillus and Bifidobacterium in acute phase and improvement phase of P. aeruginosa VAP patients. Pseudomonas (A) showed a decreased trend while Lactobacillus (B) and Bifidobacterium (C) showed an increased trend when the P. aeruginosa VAP patients obtained clinical improvement. Figure S6. The LRT microbiota of survivors of P. aeruginosa VAP patients had no significant difference compared to that of the non-survivors. Samples from day1 were collected for analysis [file 12931_2018_847_MOESM1_ESM.zip › Additional file 1 Table S6.docx]

Table S6. Negative correlation between genus of *P. aeruginosa* VAP and SOFA score

| Genus | R | P |
| --- | --- | --- |
| WCHB1-69_norank | -0.364 | 0.02890 |
| Sphingopyxis | -0.358 | 0.03189 |
| Reyranella | -0.354 | 0.03409 |
| Sphingobacterium | -0.340 | 0.04260 |
|  |  |  |
